# His Late Majesty as Hospital Patron

**Published:** 1910-05-14

**Authors:** 


### KING EDWARD VII. AND CHARITY.

### HIS LATE MAJESTY AS HOSPITAL PATRON.

At those Potsdammer dinners where they framed new systems of religion, overthrew existing social conditions, and built up in theory better ones, it was possible that Frederick the Great and his two opposing philosophers, the plagiaristic Voltaire and the conservative Maupertius, discussed the abuse of charity and devised schemes for the improvement of the poor. No authentic record of these debates, so far as this subject is concerned, has come down to us, but we have the imaginative discussion transcribed by the Austrian novelist, who makes Maupertius give utterance to the praiseworthy truism that a king must know what his subjects suffer in order to prevent such suffering. Another and a greater writer has created the fine character of Queen Louise in *The School for Princes*, eagerly investigating the social conditions of her subjects in order to amend them, wishing

> To know what life and living means and pay
> My price for being woman—bear my share
> Of suffering.*

There are few royalties who go through to the bitter end with their investigations, who pay their price for being human, and find out what suffering means, how the poor live, and how their lives may be made happier by improving their environment. There are few, indeed, who went so deeply into these questions as King Edward VII. did, and who made such good use of their experience to help and amend. It is possible that the public does not know, does not fully realise the extent to which the late King followed Maupertius' counsel. Those who wish to know how thoroughly his late Majesty interested himself in social and charitable questions cannot do better than glance over the account in *Prince, Princess, and People*,† which contains much information regarding the public activities of King Edward and Queen Alexandra. Mr. Bodley has this week put on record the good work that the late King did as member of the Commission on the Housing of the Poor, and there are many others who were privileged to have been associated with King Edward, both as King and as Prince of Wales, in charitable work, who can largely amplify the record.

Our present purpose here is merely to show, briefly and necessarily incompletely, for it is difficult to collate all the evidence from the scattered journals in which it appears, how largely his late Majesty interested himself in charitable work. He was connected, as patron, not only with many hospitals but with associations and societies of all kinds, and not only in England but throughout his wide Empire.

### The King's Hospitals.

While interesting himself in all hosqitals and contributing largely to their funds, King Edward was connected as chief patron with many institutions in a specially close and active manner. The mere list of such charities proves how far-reaching his sympathies were. In London he was patron and life governor of the large "Royal Hospitals," and of Guy's and St. Mary's (of both of which the present King is President), and in addition of the following general and special hospitals:—

Charing Cross; City of London for Diseases of the Chest; Hospital for Women and Children, Shadwell; Evelina Children's Hospital; Great Ormond Street Hospital for Children; the French Hospital; the German Hospital, Dalston; Home Hospitals Associations, Fitzroy Square; Italian Hospital; King's College Hospital; University College Hospital; London Fever Hospital; London Lock Hospital; Metropolitan Hospital; Miller General Hospital; Hospital for Paralysed and Epileptics, Queen Square; Royal Dental Hospital; Royal Ear Hospital; Royal Eye Hospital; Royal Free Hospital; Royal Hospital for Diseases of the Chest; Royal London Ophthalmic Hospital; Royal National Orthopædic Hospital; Royal Waterloo Hospital for Women and Children; Royal Westminster Ophthalmic Hospital; St. George's Hospital; Seamen's Hospital (Dreadnought); West London Hospital; Westminster Hospital; Royal General Dispensary; Royal South London Dispensary; Westminster General Dispensary; Birmingham and Midland Ear and Throat Hospital; Birmingham General Hospital; Royal National Sanatorium, Bournemouth; Royal Alexandra Hospital, Brighton; Sussex County Hospital; Bristol Royal Hospital for Sick Children; Royal Albert Hospital, Devonport; Dorset County Hospital; Princess Alice Memorial Hospital, Eastbourne; Royal Surrey County Hospital; West Norfolk and Lynn Hospital; Manchester Royal Infirmary; Royal Victoria Infirmary, Newcastle-on-Tyne; Margate Royal Sea Bathing Hospital; Norfolk and Norwich Hospital; Plymouth Royal Eye Infirmary; South Devon and East Cornwall Hospital; Royal Portsmouth Hospital; Preston and County of Lancaster Hospital; Queen Victoria Royal Infirmary; Royal Berkshire Hospital, Reading; Richmond Royal Hospital; Royal Isle of Wight County Hospital; Salford Royal Hospital; Ventnor Royal Hospital for Consumption; King Edward VII. Hospital for Windsor; Midhurst Sanatorium; Edinburgh Royal Hospital for Consumption; Brighton, Hove, and Preston Dispensary; Royal City of Dublin Hospital; Royal National Hospital for Consumption, Ireland; Metropolitan Convalescent Institution; Yarmouth Children's Convalescent Home; Royal Prince Alfred Hospital, Sydney; Anglo-American Hospital, Cairo; English Hospital, Paris; Albert Edward Hospital, Kolhapur, Bombay; Prince of Wales' Hospital, Benares; British Hospital, Cannes; British Ophthalmic Hospital, Jerusalem.

These by no means exhaust the list, to which many smaller dispensaries and hospitals have to be added to make it complete, but it is sufficient to show how varied were King Edward's sympathies and responsibilities as a hospital patron.

---

* *Vorstenschool*, act 1, scene 1.

† *Prince, Princess, and People*, by Henry C. Burdett. Longmans. 1889.

### King Edward's Charities.

Much longer is the list of the King's charities, other than hospitals. They include orphanages, asylums, associations, societies, and funds of all sorts, and in each case his Majesty's endorsement and patronage stamps the institution as thoroughly worthy of public support. The late King was founder and patron of the King's Fund, patron of the Metropolitan Hospital Sunday Fund, patron and sovereign of the Order of the League of Mercy, and patron of the St. John's Ambulance Association. He was patron also of the Surgical Aid Society; the Truss Society; the Missions to Seamen; Anti-Slavery and Aborigines' Protection Society; Army and Navy Pensioners' Employment Society; Benevolent Society of St. Patrick; Bethnal Green Free Library; British Orphan Asylum; British Red Cross Society; Choir Benevolent Fund; Church Lads' Brigade; Waifs and Strays; Christ's Hospital; Church of England Temperance Society; Church Schoolmasters' Benevolent Institution; City of London General Pension Society; Civil Service Benevolent Fund; Dartmouth Home for Crippled Boys; Epsom College; Friend of the Clergy Corporation; Gardeners' Royal Benevolent Institution; Goldsmiths' Benevolent Institution; Gordon Boys' Homes; Royal Caledonian Asylum; Royal Cambridge Asylum; Royal Female Orphans' Asylum; Royal General Theatrical Fund; Royal Masonic Institution for Girls; Royal Masonic Institution for Boys; Royal Merchant Seamen's Orphanage; Governesses' Benevolent Institution; Homes for Little Boys; Infant Orphan Asylum; Jews' Hospital and Orphan Asylum; Licensed Victuallers' Asylum; London Female Guardians' Society; London Mendicity Society; London Orphan Asylum; Marine Society; Metropolitan and City Police Orphanage; Metropolitan Public Gardens Association; National Association for Employment of Reserve Soldiers; National Association for Prevention of Consumption; National Benevolent Institute; National Society for Prevention of Cruelty to Children; Alexandra Orphanage Philanthropic Society; Post Office Orphan Homes; Printers' Pension and Orphan Asylum; Clerks' Benevolent Fund; Reedham Orphanage; Ragged Schools Union and Shaftesbury Society; Railway Benevolent Institution; Reformatory and Refuge Union; Refuge for the Destitute; Royal Albert Orphan Asylum; Royal Asylum of St. Anne's Society; Royal United Service Asylum for Orphans, Devonport; Liverpool Seamen's Orphan Institute; Royal Sailors' Rest and Royal Seamen's Orphan Schools, Portsmouth; Royal Orphanage, Wolverhampton; Association for Promoting the Welfare of the Blind; British and Foreign Blind Association; London Society for the Blind; Royal Blind Pension Society; Royal Normal College for the Blind; Edinburgh Royal Blind Asylum; Liverpool School for the Blind; Norwich Asylum for Indigent Blind; Association for Oral Instruction of Deaf and Dumb; British Asylum for Deaf and Dumb; Royal Association for Deaf and Dumb; Royal School for Deaf Children; Asylums for Deaf and Dumb at Birmingham, Derby, Manchester, and Swansea; Royal Hospital for Incurables, Putney; Royal Hospital for Incurables, Edinburgh; Royal Metal Trades Pension Society; Royal Military Benevolent Fund; Royal National Pension Fund for Nurses; Royal School for Daughters of Army Officers; Royal School for Naval and Marine Officers' Daughters; Royal Society for Prevention of Cruelty to Animals; Royal Society for Musicians of Great Britain; Royal Soldiers' Daughters' Homes; Sailors' Orphan Girls' Homes; Royal Shipwrecked Fishermen's Benevolent Society; Society for Improving Conditions of Labouring Classes; Society for Relief of Distress; Soldiers' and Sailors' Help Society; Society of Friends of Foreigners in Distress; Home for Lost and Starving Dogs; United Kingdom Railway Officers' and Servants' Benevolent Association; Clerks' and Drapers' Schools; Workmen and Lightermen's Royal Asylum, and many other provident, friendly, charitable and philanthropic societies and institutions.

### The King's Fund and the League of Mercy.

It is manifestly impossible within the space of a short article to deal adequately with his Majesty's multifarious charities. Those who wish a fuller account, with some details of his subscriptions and the many practical ways in which, while yet Prince of Wales, he showed his active interest in charitable work, are referred to the volume already quoted. It must be remembered that, apart from hospitals, orphanages, and asylums, his late Majesty was actively connected with a host of other institutions, many of them belonging to the services, which cannot be classed under these headings, though no one can doubt their charitable objects.

A word, however, must be said about his connection with the great funds and with the League of Mercy, though it is fitting that his work on behalf of these important organisations should form the subject of a separate article. Every institutional worker knows the history of the King's Fund and of the League. The establishment of the former, as the Prince of Wales' Hospital Fund for London, was due directly to the incentive of the late King, then Prince of Wales. How grandly the object of the Fund has been realised our readers know. Already in 1900 Lord Lister declared how greatly he had been impressed with the importance and value of the services rendered to the London hospitals by the Fund. Not the least of the benefits conferred upon hospitals by the institution of the Fund has been the moral effect it has produced upon numbers of men and women of influence and wealth who, impressed by the Prince of Wales' example, have taken an active interest in the welfare of the voluntary hospitals. "The movement at the outset," wrote an authority who has been prominently connected with the Fund since its inception, "was based upon the idea of personal service, and such service has been given ungrudgingly by an increasing number of people of all classes who have become identified with the work of the Fund." His Majesty, as Prince of Wales, set an example which it is gratifying to record was followed by many thousands of his subjects, to the lasting benefit of the London hospitals.

No less personal and successful have been the services he rendered to the League of Mercy, which was instituted by Royal Charter on March 13, 1899, and was established by the Prince of Wales with the co-operation of Queen Victoria. His late Majesty was Patron of the League and Sovereign of the Order of Mercy, and his example and precept in this direction helped to promote the organisation and to bring it to its present flourishing state. The League is responsible for the machinery whereby the humblest citizens, who are willing to contribute the smallest sums weekly in support of the voluntary hospitals, are reached. The good work it has done and is doing is incalculable, and can scarcely be adequately measured by the amount of the grants annually made by the League to the hospitals, large and increasing though that amount be. The chief value of the League, as of the great funds, is largely a moral one. It has brought the hospitals prominently before the public and it has brought home to every citizen the responsibilities which he bears and the obligations which he holds to do his share in the relief of suffering, sickness, and distress. And no one, assuredly, has had greater share in teaching this moral lesson to the public than the late King.

The main point is that King Edward, earnestly supported by Queen Alexandra, learned to know the needs of his subjects. He went amongst them, and took part in their work, and proved himself an able, energetic, and active worker. The life of one who has so many important duties to fulfil and so extended a domain of labour is arduous in the extreme, but he never shirked his responsibilities. Much of his good work was done quietly and without parade, so that the public to-day has scarcely an adequate idea of the magnitude of his services on its behalf. Yet the record of it remains, for anyone who wishes to look up. A study of these charitable labours of the late King will not only prove instructive, but it will be an encouragement for others to do their share in helping institutions and emulating, so far as is possible for them, the good work of Edward the Philanthropic.

